# The effects of secondary prevention after coronary revascularization in Taiwan

**DOI:** 10.1371/journal.pone.0215811

**Published:** 2019-05-02

**Authors:** Wen-Han Feng, Chun-Yuan Chu, Po-Chao Hsu, Wen-Hsien Lee, Ho-Ming Su, Tsung-Hsien Lin, Hsueh-Wei Yen, Wen-Chol Voon, Wen-Ter Lai, Sheng-Hsiung Sheu

**Affiliations:** 1 Division of Cardiology, Department of Internal Medicine, Kaohsiung Medical University Hospital, Kaohsiung, Taiwan; 2 Department of Internal Medicine, Municipal Ta-Tong Hospital, Kaohsiung, Taiwan; 3 Department of Internal Medicine, Faculty of Medicine, College of Medicine, Kaohsiung Medical University, Kaohsiung, Taiwan; Rutgers Robert Wood Johnson Medical School, UNITED STATES

## Abstract

**Background:**

Secondary prevention therapy for patients with coronary artery disease using an antiplatelet agent, β-blocker, renin-angiotensin system blocker (RASB), or statin plays an important role in the reduction of coronary events after coronary artery bypass grafting (CABG) surgery or percutaneous coronary intervention (PCI). We analyzed the status and effects of secondary prevention after coronary revascularization in Taiwan.

**Methods:**

This national population-based cohort study was conducted by analyzing the Longitudinal Health Insurance Database 2000 from the National Health Insurance Research Database of Taiwan. Patients who underwent CABG or PCI from 2004 to 2009 were included in the analysis. The baseline characteristics of the patients and ACC/AHA class I medication use at 12 months were analyzed. The primary endpoints were a composite of major adverse cardiac and cerebrovascular events.

**Results:**

A total of 5544 patients comprising 895 CABG and 4649 PCI patients were evaluated. CABG patients had more comorbidities and a higher rate of major adverse event during the follow-up period. However, use of antiplatelet agents and RASB at 12 months was significantly lower in CABG patients than in PCI patients (44.2% vs. 50.9% and 38.6% vs. 48.9%, both p < 0.01). Age, diabetes, and chronic kidney disease were independent risk factors while statin use was a protective factor for the primary endpoints in both PCI and CABG groups.

**Conclusion:**

There is still much room to improve class I medication use in secondary prevention for patients after revascularization in Taiwan. Statin could be an effective treatment to improve the outcomes.

## Introduction

Medical therapy is the cornerstone of coronary artery disease (CAD) therapy because coronary revascularization per se does not stop atherosclerosis progression. According to the clinical guidelines for the secondary prevention and risk reduction of CAD, several drugs are strongly recommended to improve outcomes including antiplatelet agents, β-blockers (BB), renin-angiotensin system blockers, and statin [1–2]. However, not all patients receive the recommended drugs after coronary revascularization including percutaneous coronary intervention (PCI) or coronary artery bypass grafting (CABG).

Mark et al. recently reported the performance of secondary prevention after revascularization in the USA [3]. They found that patients who receive successful coronary revascularization might not use the recommended medications for several reasons, including a belief that cardiac medications are no longer necessary once coronary stenosis has been treated with a stent or bypass surgery.

The aim of this study is to determine the status and effect of secondary prevention after coronary revascularization in Taiwan by analyzing data from the Taiwan National Health Insurance Research Database (NHIRD).

## Materials and methods

### Study patients and design

**Fig 1:**
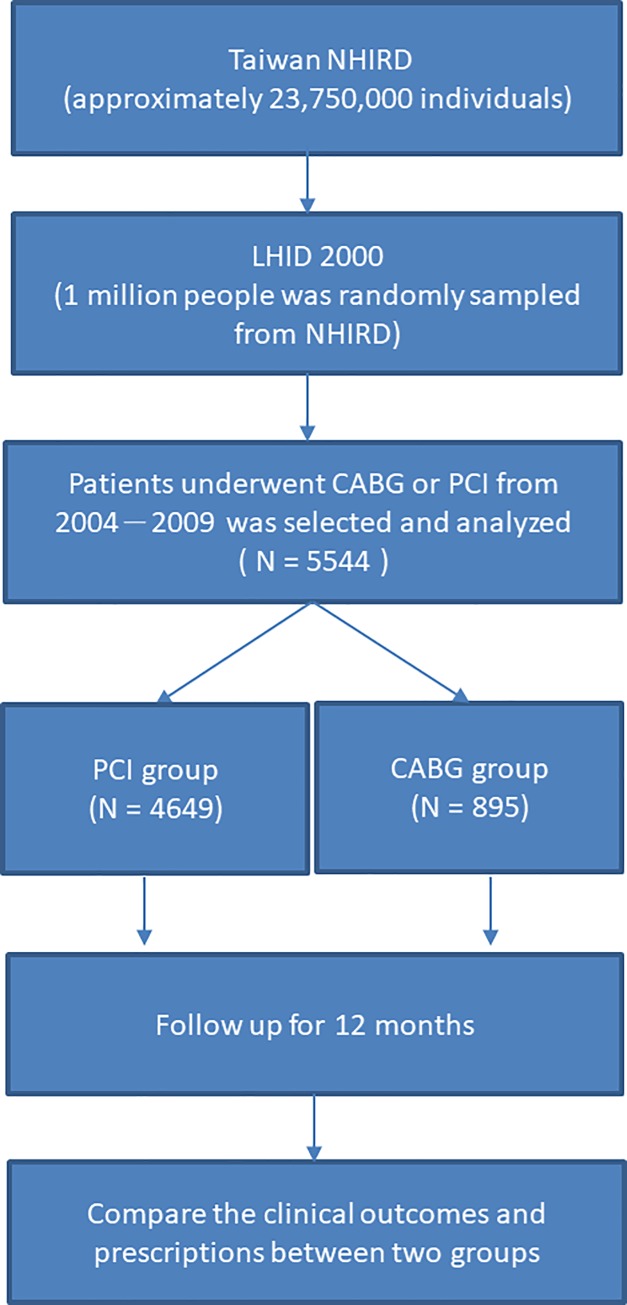
Flow chart of the study. NHIRD, National Health Insurance Research Database; LHID, Longitudinal Health Insurance database.

This population-based cohort study was conducted using data from the Longitudinal Health Insurance Database 2000 (LHID 2000) from the NHIRD of Taiwan (Fig 1). Taiwan National Health Insurance (NHI) is a single-payer national health insurance program launched on March 1, 1995, that covers 99.9% of all Taiwanese residents. LHID 2000 is a randomly sampled dataset of one million beneficiaries from the year 2000 registry of all NHI enrollees. The registry contains information for approximately 23.75 million individuals, with all registration files and original claims data for reimbursement and academic analysis [4]. According to the National Health Research Institutes, no significant differences exist in the age, sex, and healthcare costs between the sampled group and all enrollees in NHI [4].

The patient data were analyzed from the database, in which the international classification of disease-9 (ICD-9) codes is used to define the corresponding diseases. Patients who underwent CABG or PCI from January 2004 to December 2009 were selected for inclusion in the analysis. The prescriptions after the procedures were assessed to determine the kinds of drugs prescribed. Utilization of statins, BB, angiotensin converting enzyme inhibitors (ACEI), angiotensin II type 1 receptor blockers (ARB), and antiplatelets (including aspirin and clopidogrel) was determined by analyzing the prescription of these drugs within 3 months after the procedure.

### Collection of demographic data

Demographic data and cardiovascular risk factors including age, gender, hypertension, diabetes mellitus (DM), chronic kidney disease (CKD), old myocardial infarction (OMI), chronic obstructive pulmonary disease (COPD), and hyperlipidemia were obtained from the NHIRD. The profiles of prescription drugs including statin, aspirin, clopidogrel, BB, ACEI, and ARB were recorded and analyzed.

The primary endpoints of this study were a composite of major adverse cardiac and cerebrovascular events (MACCE), including all-cause mortality, myocardial infarction (MI) (ICD-9 code: 410) and unstable angina (ICD-9 code: 4111), stroke (ICD-9 code: 436), revascularization either by PCI (ICD-9 codes: 33076B, 33077B, 33078B) or CABG (ICD-9 codes: 68023B, 68024B, 68025B), and hospitalization for heart failure.

### Statistical analysis

All data were expressed as percentages or means ± standard deviations for continuous variables. Categorical variables were compared between groups using the Chi-square test. Time-to-event analysis and covariates of risk factors were modeled using the Cox proportional hazards model. The follow-up period was recorded starting on the date of diagnosis and ending on the date of the development of different outcomes or at the last observation up to December 31, 2010. Significant variables in univariate analysis were selected for multivariate analysis. A p value < 0.05 was considered statistically significant. All statistical operations were performed using the SAS software version 9.2 (SAS Institute, Cary, NC, USA).

### Ethic statement

Our study was approved by the Kaohsiung Medical University Chung-Ho Memorial Hospital Institutional Review Board (KMUHIRB-EXEMPT(I)- 20180028). Because all data from NHIRD was anonymous, informed consent was not needed.

## Results

The cohort comprised 5544 patients who received coronary revascularization. Among them, 895 underwent CABG and 4649 underwent PCI. The baseline characteristics of both groups are shown in Table 1. Patients who underwent CABG had more comorbidities including DM, hypertension, hyperlipidemia, CKD, and COPD.

**Table 1:** Baseline characteristics in CABG and PCI groups.

	CABG	PCI	p value
Total	895	4649	
Age	65.2	66.0	0.0601
Male (%)	680 (75.9)	3367 (72.4)	0.0283
Diabetes (%)	434 (48.4)	1785 (38.3)	<0.0001
Hypertension (%)	662 (73.9)	3215 (69.1)	0.0004
Hyperlipidemia (%)	382 (42.6)	1643 (35.3)	<0.0001
Chronic kidney disease (%)	104 (11.6)	389 (8.3)	0.0045
Old myocardial infarction (%)	89 (9.9)	211 (4.5)	<0.0001
Chronic obstructive pulmonary disease (%)	124 (13.8)	720 (15.4)	0.2132
Acute coronary syndrome (%)	203 (22.7)	1390 (29.9)	<0.0001
β-blocker (%)	382 (42.6)	1996 (42.9)	0.8889
Antiplatelet (%)	396 (44.2)	2368 (50.9)	0.0002
Statin (%)	362 (40.4)	1882 (40.4)	0.9845
ACEI/ARB (%)	346 (38.6)	2274 (48.9)	<0.0001
MACCE at 12 months	303 (33.9)	1059 (22.8)	<0.0001
All-cause mortality	138 (15.4)	435 (9.4)	<0.0001

PCI, percutaneous coronary intervention; CABG, coronary artery bypass grafting; ACEI, angiotensin converting enzyme inhibitors; ARB, angiotensin II type 1 receptor blockers; MACCE, major adverse cardiac and cerebrovascular event

The medications used and the incidence of MACCE at 12 months in both groups are also presented in Table 1. Patients in the CABG group experienced more cardiovascular events at 12 months. There was no statistically significant difference in BB and statins use between the two groups but more patients in the PCI group had taken ACEI/ARB and antiplatelets at the 12th month than patients in the CABG group. Those still taking antiplatelets had more cases of hyperlipidemia and prior PCI but less history of OMI and COPD (Table 2). Those still taking BB had more cases of hypertension, hyperlipidemia, and PTCA history but less COPD (Table 3). Those still taking antiplatelets had more cases of hyperlipidemia but less CKD and COPD (Table 4). Those still taking ACEI/ARB had more cases of diabetes, hypertension, and PCI history but less CKD and CABG (Table 5).

**Table 2:** Factors associated with antiplatelet use at 12 months.

	Antiplatelet Use	No Antiplatelet Use	p value
Total (%)	2764 (49.9)	2780 (50.1)	
Age	65.6 ± 11.7	66.3 ± 12.4	0.0499
Male (%)	2025 (73.3)	2022 (72.7)	0.6570
Diabetes (%)	1125 (40.7)	1094 (39.4)	0.3052
Hypertension (%)	1940 (70.2)	1937 (69.7)	0.6777
Hyperlipidemia (%)	1050 (38.0)	975 (35.1)	0.0241
CKD (%)	225 (8.1)	253 (9.1)	0.2028
Old MI (%)	141 (5.1)	159 (5.7)	0.3091
COPD (%)	387 (14.0)	457 (16.4)	0.0115
CABG (%)	396 (14.3)	499 (17.9)	0.0002
PCI (%)	2490 (90.1)	2371 (85.3)	<0.0001

CKD, chronic kidney disease; MI, myocardial infarction; COPD, chronic obstructive pulmonary disease; CABG, coronary artery bypass grafting; PCI, percutaneous coronary intervention

**Table 3:** Factors associated with β-blocker use at 12 months.

	β-blocker Use	No β-blocker Use	p value
Total (%)	2378 (42.9)	3166 (57.1)	
Age	63.9 ± 11.7	67.4 ± 12.1	<0.0001
Male (%)	1756 (73.8)	2291 (72.4)	0.2190
Diabetes (%)	963 (40.5)	1256 (39.7)	0.5351
Hypertension (%)	1752 (73.7)	2125 (67.1)	<0.0001
Hyperlipidemia (%)	968 (40.7)	1057 (33.4)	<0.0001
CKD (%)	194 (8.2)	284 (9.0)	0.2863
Old MI (%)	125 (5.3)	175 (5.5)	0.6590
COPD (%)	277(11.6)	567 (17.9)	<0.0001
CABG (%)	382(16.1)	513 (16.2)	0.8889
PCI (%)	2111 (88.8)	2750 (86.9)	0.0321

CKD, chronic kidney disease; MI, myocardial infarction; COPD, chronic obstructive pulmonary disease; CABG, coronary artery bypass grafting; PCI, percutaneous coronary intervention

**Table 4:** Factors associated with statin use at 12 months.

	Statin Use	No Statin Use	p value
Total (%)	2244 (40.5)	3300 (59.5)	
Age	63.4 ± 11.6	67.6 ± 12.1	<0.0001
Male (%)	1649 (73.5)	2398 (72.7)	0.5006
Diabetes (%)	899 (40.1)	1320 (40.0)	0.9629
Hypertension (%)	1544 (68.8)	2333 (70.7)	0.1317
Hyperlipidemia (%)	1079 (48.1)	946 (28.7)	<0.0001
CKD (%)	137 (6.1)	341 (10.3)	<0.0001
Old MI (%)	109 (4.9)	284 (8.6)	0.0810
COPD (%)	271 (12.1)	573 (17.4)	<0.0001
CABG (%)	362 (16.1)	533 (16.2)	0.9845
PCI (%)	1978 (88.1)	2883 (87.4)	0.3842

CKD, chronic kidney disease; MI, myocardial infarction; COPD, chronic obstructive pulmonary disease; CABG, coronary artery bypass grafting; PCI, percutaneous coronary intervention

**Table 5:** Factors associated with ACEI/ARB use at 12 months.

	ACEI/ARB Use	No ACEI/ARB Use	p value
Total (%)	2620 (47.3)	2924 (52.7)	
Age	65.5 ± 11.9	66.2 ± 12.2	0.0214
Male (%)	1882 (71.8)	2165 (74.0)	0.0642
Diabetes (%)	1134 (43.3)	1085 (37.1)	<0.0001
Hypertension (%)	2005 (76.5)	1872 (64.0)	<0.0001
Hyperlipidemia (%)	982 (37.5)	1043 (35.7)	0.1622
CKD (%)	167 (6.4)	311 (10.6)	<0.0001
Old MI (%)	143 (5.5)	157 (5.4)	0.8842
COPD (%)	395 (15.1)	449 (15.4)	0.7725
CABG (%)	346 (13.2)	549 (18.8)	<0.0001
PCI (%)	2372 (90.5)	2489 (85.1)	<0.0001

CKD, chronic kidney disease; MI, myocardial infarction; COPD, chronic obstructive pulmonary disease; CABG, coronary artery bypass grafting; PCI, percutaneous coronary intervention

Cox regression analysis showed that age, diabetes, CKD, and COPD were risk factors while male sex and statin use were protective factors for the primary endpoints in the PCI group (Table 6). The hazard ratios (95% confidence interval) were 1.03 (1.02–1.04), 1.49 (1.31–1.69), 1.66 (1.39–1.99), 1.19 (1.02–1.39), 0.87 (0.76–0.99), and 0.74 (0.64–0.85), respectively. In the CABG group (Table 7), age, diabetes, and CKD were risk factors and statin use was a protective factor for the primary endpoints. The hazard ratios (95% confidence interval) were 1.03 (1.02–1.04), 1.34 (1.06–1.70), 1.59 (1.16–2.18), and 0.76 (0.58–1.00), respectively.

**Table 6:** Cox regression analysis in PCI group.

variable	HR	lower 95%CI	upper 95%CI	p value
Age	1.03	1.02	1.04	<0.0001
Male	0.87	0.76	0.99	0.0347
Diabetes	1.49	1.31	1.69	<0.0001
Hypertension	0.94	0.82	1.09	0.4051
CKD	1.66	1.39	1.99	<0.0001
Old MI	0.80	0.60	1.07	0.1259
COPD	1.19	1.02	1.39	0.0293
ACEI/ARB	0.98	0.86	1.12	0.7286
β-blocker	0.99	0.86	1.13	0.8383
Statin	0.74	0.64	0.85	<0.0001
Antiplatelet	0.89	0.79	1.02	0.0899

PCI, percutaneous coronary intervention; CKD, chronic kidney disease; MI, myocardial infarction; COPD, chronic obstructive pulmonary disease; ACEI, angiotensin converting enzyme inhibitors; ARB, angiotensin II type 1 receptor blockers

**Table 7:** Cox regression analysis in CABG group.

variable	HR	lower 95%CI	upper 95%CI	P-value
Age	1.03	1.02	1.04	<0.0001
Male	1.27	0.96	1.68	0.0947
Diabetes	1.34	1.06	1.70	0.0152
Hypertension	0.88	0.67	1.16	0.3589
CKD	1.59	1.16	2.18	0.0042
Old MI	1.04	0.72	1.50	0.8302
COPD	1.08	0.80	1.46	0.6291
ACEI/ARB	1.12	0.88	1.43	0.3635
β-blocker	0.98	0.75	1.27	0.8577
Statin	0.76	0.58	1.00	0.0491
Antiplatelet	0.86	0.68	1.11	0.2440

CABG, coronary artery bypass grafting; CKD, chronic kidney disease; MI, myocardial infarction; COPD, chronic obstructive pulmonary disease; ACEI, angiotensin converting enzyme inhibitors; ARB, angiotensin II type 1 receptor blockers

**Fig 2:**
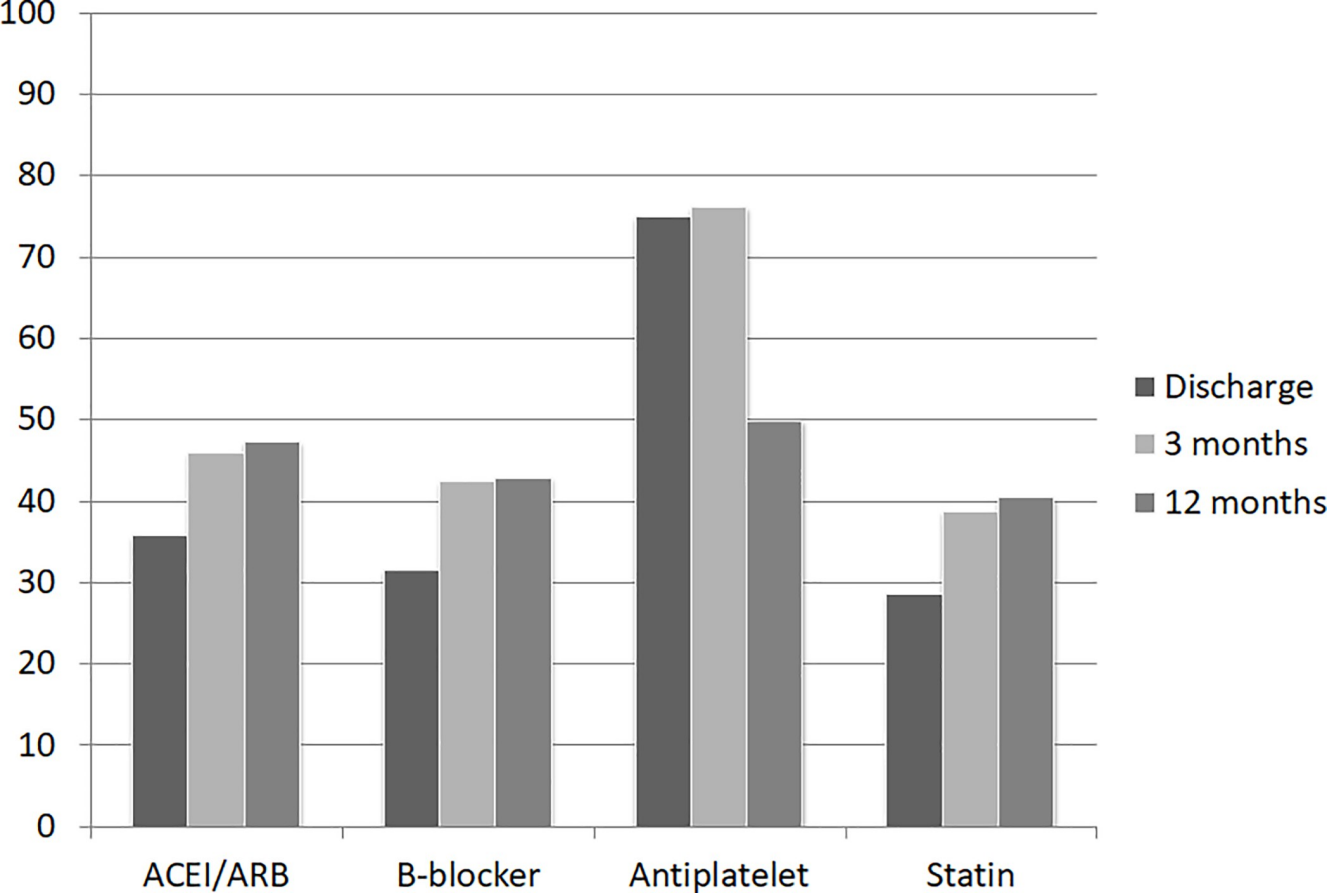
Trend in prescription changes over 1 year.

**Fig 3:**
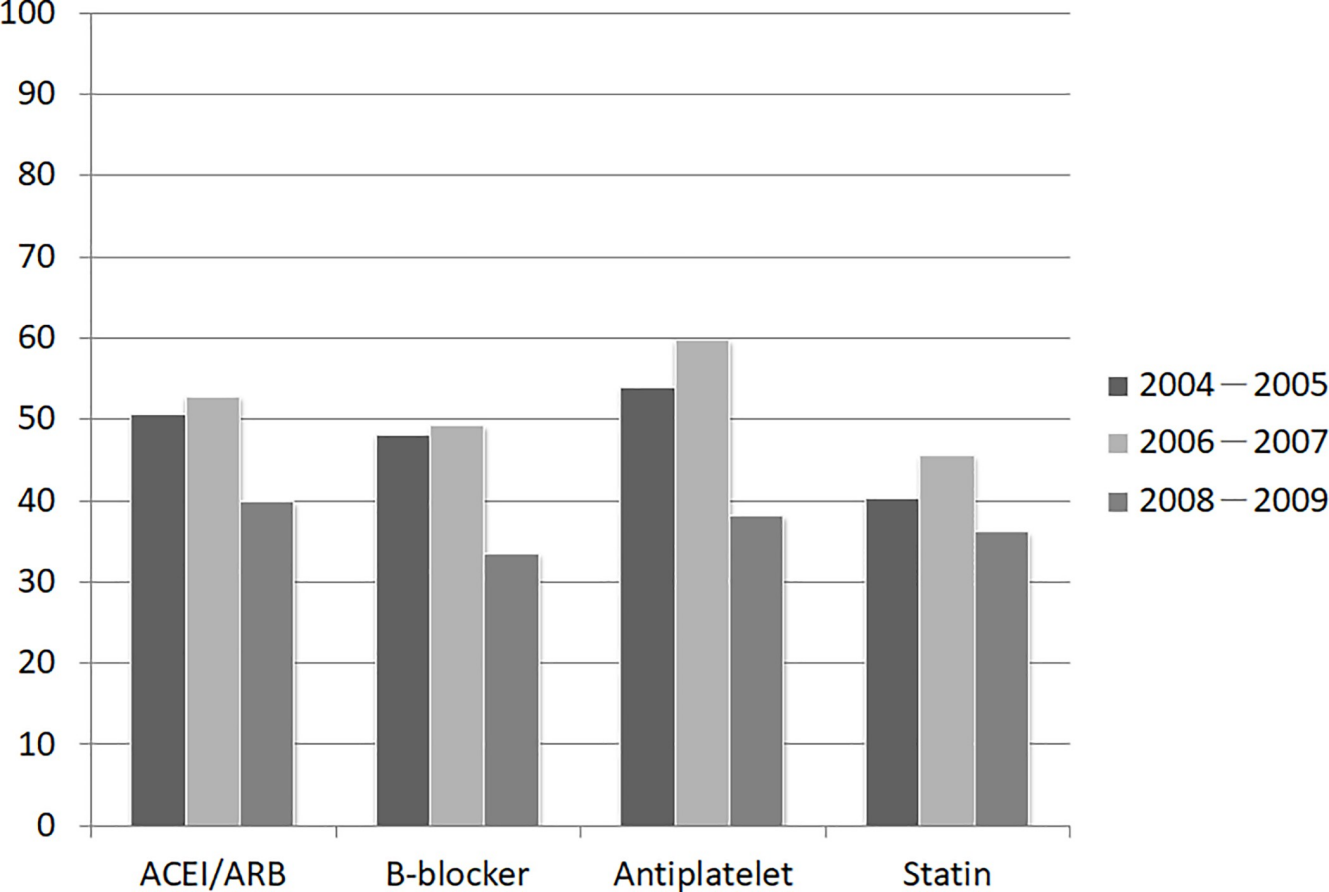
Trend in prescription changes during the follow-up years.

The trends in prescription changes for these medications after 1 year and during the follow-up years i.e., from 2004 to 2009 were also analyzed (Tables 8 and 9). The use of all medications except antiplatelet increased from the time of discharge to the 12-month follow-up (Fig 2). Patients were divided into three groups according to the time they received PCI or CABG: 1) 2004–2005, 2) 2006–2007, and 3) 2008–2009. (Fig 3).

**Table 8:** Trend in prescription changes over 1 year.

	Discharge	3 months	12 months
ACEI/ARB (%)	2169 (39.1)	2549 (45.9)	2620 (47.3)
β-blocker (%)	1773 (32.0)	2351 (42.4)	2378 (42.9)
Antiplatelet (%)	4157 (74.9)	4221 (76.1)	2764 (49.9)
Statin (%)	1811 (32.6)	2155 (38.9)	2244 (40.5)

ACEI, angiotensin converting enzyme inhibitors; ARB, angiotensin II type 1 receptor blockers

**Table 9:** Trend in prescription changes during the follow-up years.

	PCI/CABG in 2004–2005 (N = 1531)	PCI/CABG in 2006–2007 (N = 1887)	PCI/CABG in 2008–2009 (N = 2126)
ACEI/ARB (%)	776 (50.7)	997 (52.8)	847 (39.8)
β-blocker (%)	737 (48.1)	929 (49.2)	712 (33.5)
Antiplatelet (%)	827 (54.0)	1127 (59.7)	810 (38.1)
Statin (%)	617 (40.3)	859 (45.5)	768 (36.1)

ACEI, angiotensin converting enzyme inhibitors; ARB, angiotensin II type 1 receptor blockers

A total of 573 patients died during the follow-up period. These patients had a significantly lower percentage of medication use compared with the patients who survived (Table 10). We also analyze the use of each medication at discharge and the rate of death and recurrent myocardial infarction during the period of follow-up (Table 11). We found that the rate was higher in the patients who were not taking these drugs at discharge. (20.4% in patients without antiplatelet vs 15.5% in those with antiplatelet, 18.1% in those without ACEI/ARB vs 14.6% in those with ACEI/ARB, 19.0% in those without statin vs 12.0% in those with statin, 18.2% in those without β-blocker vs 13.6% in those with β-blocker)

**Table 10:** The mortality of patients with and without prescription medication at the end of follow-up.

		Death	Live	p value
	Total	N(%)	N (%)	
Total	5544	573 (10.3)	4971 (89.7)	
ACEI/ARB	2549	146 (25.5)	2403 (48.3)	<0.0001
β-blocker	2351	118 (20.6)	2233 (44.9)	<0.0001
Antiplatelet	4221	275 (48.0)	3946 (79.4)	<0.0001
Statin	2155	86 (15.0)	2069 (41.6)	<0.0001

ACEI, angiotensin converting enzyme inhibitors; ARB, angiotensin II type 1 receptor blockers

**Table 11:** The rate of death and recurrent myocardial infarction in patients with/without the medication in their prescription at discharge.

	Total	Antiplatelet -	Antiplatelet +	ACEI/ARB -	ACEI/ARB +	Statin -	Statin +	β-blocker -	β-blocker +
Total	5544	1387	4156	3375	2169	3733	1811	3771	1773
Death/Re-MI (%)	927 (16.7)	284 (20.4)	643 (15.5)	611 (18.1)	316 (14.6)	710 (19.0)	217 (12.0)	686 (18.2)	241 (13.6)

Re-MI, recurrent myocardial infarction; ACEI, angiotensin converting enzyme inhibitors; ARB, angiotensin II type 1 receptor blockers

## Discussion

There were three major findings in this study. First, evidence-based medicine was still being applied for less than 50% of patients who received revascularization including PCI and CABG at the 12^th^ month in Taiwan. Second, patients who took statin continuously had fewer cardiovascular events in both the PCI and CABG groups. Third, DM and CKD were risk factors for the primary endpoints in both groups.

Possible reasons for the cessation of evidence-based therapies could be inertia on the part of the patients or physicians. Patients might misconceive PCI or CABG as the definitive treatment for CAD and therefore stop taking their medicine. Furthermore, they may think that CAD is no longer severe enough after revascularization to require medication. The prescription rate in our study was relatively low compared with that in studies performed in other countries. One study in the USA reported that around 70% of patients continued to use ACE/ARB, statin, and BB after PCI or CABG [3]. Another study reported a medication adherence of 67.3% for antiplatelets, 69.7% for BB, 32.5% for ACEI/ARB, and 61.3% for statin one year after CABG [5]. A study in Germany showed the medication use a year after acute MI was 73.7% for aspirin, 72.5% for statin, 70% for ACEI, and 80% for BB [6]. There are huge gaps between current guideline recommendations and real-world practice in Taiwan. More work including physician education is needed to encourage the use of evidence-based medicine if no contraindications exist.

The drugs we investigated have been widely demonstrated to have benefits for the secondary prevention of atherosclerotic cardiovascular disease. Aspirin can reduce the risk of cardiovascular death and MI by 25% [7]. Dual antiplatelet therapy has also been proven to have more benefits than aspirin alone in patients who received PCI with stent placement [8]. ACEI can reduce the rate of cardiovascular events particularly among patients with left ventricular dysfunction, DM, hypertension, and CKD [9]. BBs have been proven to be beneficial in patients with left ventricular dysfunction, acute coronary syndrome, or prior MI. Furthermore, BBs also reduces the incidence of postoperative atrial fibrillation in CABG patients. Therefore, current guidelines recommend BBs for all CABG patients at the time of hospital discharge. Statin is the most evidence-based medicine available to treat dyslipidemia and should be prescribed to all patients in the absence of contraindications. The ASCOT-LLA and WOSCOPS trials demonstrate the long-term benefits of lowering LDL-C for the reduction of cardiovascular diseases [10–11]. In our study, statin treatment had cardiovascular benefits for both the PCI and CABG groups. Factors associated with lower 12-month statin use including age, CKD, DM, and COPD should be taken into consideration in daily practice.

DM is a strong risk factor for cardiovascular complications as it increases the risk of greater severity and progression of coronary disease and should be treated aggressively. Patients with CKD are at a higher risk for cardiovascular disease, and particular care should be taken to address comorbidities and disease management [12]. Patients with COPD are also at risk for cardiovascular events, which may be attributed to increased systemic inflammation. In our study, we found that patients with DM or CKD had a higher risk of further cardiovascular events in both the PCI and CABG groups. COPD was also found to be an independent risk factor for cardiovascular events in the PCI group. Patients with DM or CKD are recognized as a very high-risk group in current guidelines and need to receive intensive treatment to lower their risk for future cardiovascular events [13]. A previous study found that the exacerbation of COPD increases the risk of MI and stroke [14–15]. Our study provides more evidence of the importance of paying more attention to patients with CKD, DM, and COPD to reduce future cardiovascular events after coronary revascularization.

Patients who died during the follow-up period received much fewer medications than those who survived. One possible reason is that the condition of these patients was not suitable for the medications (e.g., bleeding complications, hypotension, acute kidney injury, etc.), and the doctor stopped the medication prematurely. However, the drugs we investigated have been widely demonstrated to have benefits for the secondary prevention of atherosclerotic cardiovascular disease. Furthermore, the prescription at discharge seems to be important for the patients and has some impact for the clinical outcomes. Every effort should be made to encourage prescription especially before discharge.

This retrospective study has several limitations. First, we lacked sufficient detailed clinical data to explore the reasons why certain medications were not prescribed, such as information on the serum creatinine level, left ventricular function, and LDL level, which could have affected the prescription pattern. Second, our data only showed the drugs prescribed by doctors, but whether the patients actually took the medications remains unknown and this might influence the effect of the drugs on the cardiovascular outcomes. Third, the underlying diseases were determined based on the ICD codes. We may have underestimated the patients’ comorbidities for cases in which the doctors did not record the codes. Fourth, the national insurance reimbursement criteria may limit the medications physicians can prescribe to meet the clinical practice guidelines. Fifth, there could be some survivor bias while assessing for medication usage and hazards of cardiac outcomes. However, the drugs we investigated have been widely demonstrated to have benefits for the secondary prevention of atherosclerotic cardiovascular disease. Sixth, the national health insurance only allowed 9-month dual antiplatelet treatment which might partially explain the significant reduction of antiplatelet from 9^th^ month to 12^th^ month.

## Conclusions

Much room for improvement in daily practice remains for the secondary prevention of CAD after revascularization in Taiwan. Statin is the most important of 4 ACC/AHA Class I drugs that can improve the outcomes. Physicians should be encouraged to prescribe statin if no contraindication. The benefit of long-term use of 4 ACC/AHA Class I drugs should be educated to all physicians. Furthermore, the control of risk factors including DM, CKD, and COPD is important in Taiwan. Further research is needed to understand the reasons evidence-based medications are not prescribed after CABG or PCI, and to develop appropriate strategies to improve prescription.
